# Phylogenetic signal in tooth wear dietary niche proxies: What it means for those in the field

**DOI:** 10.1002/ece3.4540

**Published:** 2018-10-09

**Authors:** Danielle Fraser, Ryan J. Haupt, W. Andrew Barr

**Affiliations:** ^1^ Canadian Museum of Nature Palaeobiology Ottawa ON Canada; ^2^ Department of Paleobiology Smithsonian Institution, National Museum of Natural History District of Columbia Washington; ^3^ Department of Geology and Geophysics University of Wyoming Laramie Wyoming; ^4^ Center for the Advanced Study of Human Paleobiology, Department of Anthropology George Washington University District of Columbia Washington

**Keywords:** mesowear, microwear, phylogenetic signal, tooth wear

## Abstract

In response to DeSantis et al., we describe that the presence of phylogenetic signal in tooth wear dietary niche proxies is likely a result of the evolutionary process. We also address their concerns regarding enforcement of the use of phylogenetic comparative methods by editors of ecology and evolution journals.

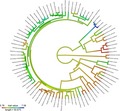

In our recent study, we describe strong phylogenetic signal in tooth wear dietary proxies. We believe that phylogenetic signal is detectible in tooth wear because the feeding apparatus acts as an evolutionary and functional module (Fraser & Rybczynski, 2014). Due to the significant functional role of the feeding apparatus, changes in one component (e.g., masseter muscle orientation) impact the other components of the module (e.g., tooth cusp relief). The result is a difference in chewing stroke, the ways in which the teeth contact each other, and, thus, the ways which the teeth wear. We further argue that such modularity of the chewing apparatus results in certain functional and thus evolutionary constraints (e.g., hypsodonty in *Equus*), potentially leading to phylogenetic niche conservatism (PNC; although it was beyond the scope of our study to perform definitive tests for niche conservatism). The end result is that tooth wear (like diet) shows strong phylogenetic signal. In fact, our finding seems unsurprising given that much of the methodological practice in the application of analytical tooth wear methods acknowledges this phylogenetic signal (e.g., by selecting appropriate teeth and wear facets specific to those chosen teeth for analysis based on functional knowledge of tooth occlusion (Ungar et al., 2010) or limiting comparisons to closely‐related taxa; e.g., Haupt, DeSantis, Green, & Ungar, 2013; DeSantis & Haupt, 2014). We subsequently make a recommendation that, when possible and necessary, appropriate phylogenetic comparative methods (PCMs) be applied, a recommendation that has been made time and time again in the ecology and evolution literature (Barr & Scott, 2014; Blomberg & Garland, 2002; Cooper, Jetz, & Freckleton, 2010; Freckleton, Harvey, & Pagel, 2002; Garland, Harvey, & Ives, 1992; Martins, 2000; Martins & Hansen, 1997; Pagel, 1992, 1999 ; Price, 1997). DeSantis et al. (2018), however, express concern that (a) our study will cause editors to unnecessarily enforce the use of phylogenetic comparative methods during review and (b) that our study will mislead researchers into thinking phylogenetic signal is merely an artifact, rather than a result of the evolutionary process.

In our recent study, we do not suggest that tests for phylogenetic signal and PCMs be applied when they are unnecessary. For example, studies focused on a small number of species within the same genus are unlikely to require such statistical corrections (although we would recommend testing for phylogenetic signal using one of the many available tests) (Harmon, Weir, Brock, Glor, & Challenger, 2008; Revell, 2011). We also recommend authors be familiar with the limitations of tests for phylogenetic signal when their sample of species is small (Münkemüller et al., 2012). Similarly, comparisons among individuals within the same species obviously do not require phylogenetic signal correction. Therefore, we recommend the use of PCMs when the phylogenetic and taxonomic breadth of a study is large (e.g., sampling camelids and equids) (Fraser & Rybczynski, 2014; Fraser, Zybutz, Lightner, & Theodor, 2014). There is an extensive literature on the statistical difficulties stemming from significant phylogenetic signal (Felsenstein, 1985; Freckleton et al., 2002; Garland et al., 1992; Garland, Bennett, & Rezende, 2005; Price, 1997). We reiterate that statistical methods make a variety of assumptions about the input data. An ordinary least squares regression, for example, makes the assumption that the residuals of y ~ x are not auto‐correlated. Phylogenetic signal is a type of autocorrelation or nonindependence among data points or residual error of y from a linear regression (Felsenstein, 1985; Revell, 2010). When traits evolve along phylogenetic lineages, the assumption of independence is violated, and thus, p‐values may be misleading and false‐positives may occur) (Barr & Scott, 2014; Rohlf & Hansen, 2006). Therefore, high Type I error rates are well documented for analyses of datasets with high phylogenetic signal in the ecology and evolution literature. There is nothing fundamentally unusual about tooth wear data as compared to other types of interspecific comparative data. The statistical question at the heart of many tooth‐wear studies is: “how strongly is the tooth‐wear proxy variable correlated with our measure of diet?” This is no different from the statistical question at the heart of any other correlational study in which the use of PCMs would be widely accepted. We therefore doubt that our study will significantly impact the decisions of editors for academic journals in ecology and evolution; most are already aware of phylogenetic signal and its statistical consequences. We have added to that vast literature an interesting observation that tooth wear may not in fact be “taxon‐free” and we do hope that editors will question (to a reasonable extent) this assertion in future studies of mammalian tooth wear. It is the onus of individual authors to determine and debate with individual editors for or against the use of PCMs, as DeSantis et al. (2018) indicate they have done successfully in the recent past.

As made clear in our recent study, we believe that the phylogenetic signal in dental wear proxies is no mere artifact: we believe it stems directly from the phylogenetic signal found in diet and the tendency for descendant species to share dietary preferences (and thus tooth‐wear patterns) with their ancestors. Our original study explicitly draws this connection, and most of the Discussion section is devoted to this point. However, phylogenetic signal is a statistical pattern that does not describe process but only the statistical nonindependence of species’ trait values (Revell, Harmon, & Collar, 2008). We strongly assert that the source of phylogenetic signal in diet and thus tooth wear is the evolutionary process of decent with modification from a common ancestor. However, there is a complex relationship among evolutionary process, rate, and phylogenetic signal (Revell et al., 2008). The implication of the DeSantis et al.’s argument, however, is that if phylogenetic signal results from the evolutionary process, there is no need to correct for it statistically. We disagree strongly with this implication. Even if the phylogenetic signal in dietary proxies such as mesowear and microwear is “meaningful” in the sense that it is related to diet, this does not change the statistical difficulties introduced by phylogenetic nonindependence. Furthermore, in no way does the presence of phylogenetic signal, nor suggesting that PCMs be used when necessary, imply that dietary proxies are unreliable indicators of diet.

Suppose we are using hypsodonty (presence of high crowned teeth) as a metric for inferring grazing behavior among mammals (let us put aside the well‐documented relationship between hypsodonty and dietary grit for the sake of this argument, which we intend only as a demonstration of phylogenetic signal) (Damuth & Janis, 2011; Jardine, Janis, Sahney, & Benton, 2012). If we sample a number of mammals and use a discriminant function analysis to estimate the strength of association between hypsodonty and feeding behavior, we would find that hypsodonty is a very good indicator of a grazing lifestyle. However, the gelada baboon, an open‐area feeder whose diet is dominated by grasses (Fashing, Nguyen, Venkataraman, & Kerby, 2014), would be misclassified by the discriminant function, based on the of absence of hypsodont cheek teeth. Thus, the strength of the association between hypsodonty and grazing is over‐estimated in this scenario. Hypsodonty is an honest indicator of grazing (Damuth & Janis, 2011; Jardine et al., 2012), but it is constrained to certain phylogenetic groups, and there exist other ways to be a grazer that do not require hypsodonty.

In a further hypothetical example used by DeSantis et al. (2018), families on their way to the airport tend to be either “knife carrying” or “knifeless.” We extend this example to suggest that, if the material from which the knives are made shows “phylogenetic signal” (e.g., some families carry ceramic knives that cannot be detected by magnetometers and others metal knives that can), airport security might overestimate the strength of the link between beeping magnetometer alarm and the presence of sharp cutting tools. While the beeping alarm is an honest signal of the presence of a knife, there are other equivalent knife‐wielders that are not detectable by this method. In our recent study, we use a phylogenetic discriminant function analysis to demonstrate the result of such overconfidence arising from phylogenetic signal on our ability to correctly classify mammals by diet using tooth wear (Fraser, Haupt, & Barr, 2018).

DeSantis et al. (2018) further suggest that (a) convergence of diets among distantly related mammals and (b) intraspecific dietary differences negate the need to correct for phylogenetic signal in tooth wear. We consider the following example. Hypsodonty has evolved numerous times within Mammalia but; for our purposes, let us consider only artiodactyls and perissodactyls. All extant equids (e.g., zebras and asses) are hypsodont. Some clades of artiodactyls possess similarly high‐crowned teeth such as members of the tribe Bovini and family Antilocapridae. The presence of high‐crowned teeth is notably nonrandomly distributed throughout Artiodactyla and Perissodactyla (Figure 1). Hypsodont members of these clades have inherited their high‐crowned teeth from a common ancestor. In fact, as far as we are aware, hypsodonty never reverses; hypsodont taxa never return to a low‐crowned condition. So, we would attain significant estimates of phylogenetic signal for hypsodonty even though it has arisen multiple times via different developmental and genetic mechanisms. Convergent evolution therefore does not negate the need for phylogenetic comparative methods.

**Figure 1:**
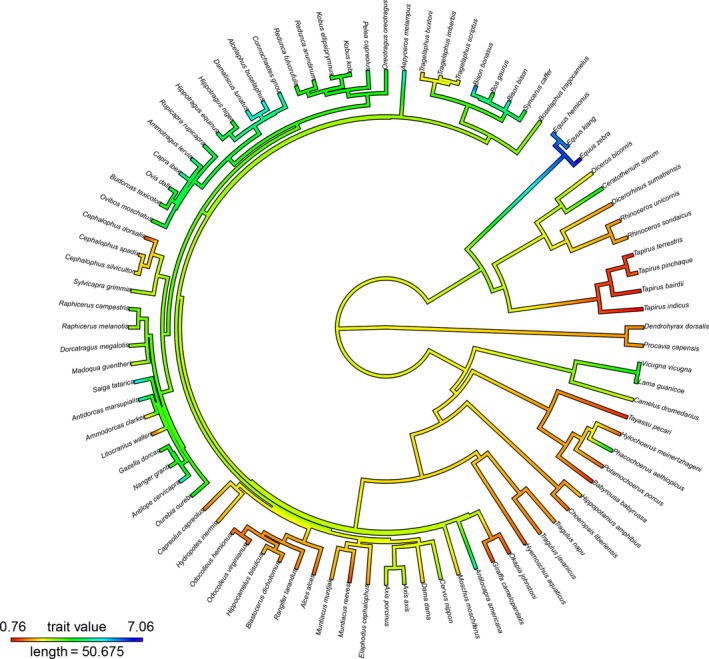
Hypsodonty shows phylogenetic patterning among hoofed mammals. Phylogenetic trait map of hypsodonty index. Blue indicates the highest hypsodonty indices while red indicates the lowest. λ = 0.97 as estimated using the fitContinuous function in the geiger R package (Harmon et al., 2008). Data are derived from Mendoza et al. (2002)

Dietary change through time might indicate weak or absent PNC. However, as acknowledged by DeSantis et al. (2018), PNC, and phylogenetic signal are not the same phenomenon (Münkemüller, Boucher, Thuiller, & Lavergne, 2015; Revell et al., 2008). Significant phylogenetic signal can result both from a Brownian Motion model of evolution, wherein a trait evolves via a random walk (Felsenstein, 1985), as well as PNC, a phenomenon wherein closely related species are more similar than is expected given a Brownian Motion model of trait evolution (Losos, 2008). Therefore, significant phylogenetic signal can occur in the absence of PNC (although we doubt that insignificant phylogenetic signal is likely to occur in the presence of significant PNC) (Revell et al., 2008). The fact that intraspecific shifts in diet occur among mammals therefore has no bearing on whether dietary phylogenetic signal exists within Mammalia or whether it needs to be corrected for. The tooth wear of members of a species can and do vary through time (Calandra & Merceron, 2016; DeSantis et al., 2017; DeSantis & Haupt, 2014; Rivals, Mihlbachler, & Solounias, 2007; Rivals, Schulz, & Kaiser, 2008; Rivals & Semprebon, 2006) but we suggest that they vary within some range (i.e., the fundamental rather than realized niche) that is determined by functional constraints of the feeding apparatus. This manifests as dietary shifts, say from a less gritty diet to a more gritty diet, but should rarely manifest as an intraspecific shift from herbivory to carnivory, for example (Price, Hopkins, Smith, & Roth, 2012). We further agree with DeSantis et al. (2018) that experimental diets can and do change the properties of mammal tooth wear (Hoffman, Fraser, & Clementz, 2015). However, our study refers only to the natural diet of mammals. Feeding a lion rocks would most assuredly change how the teeth wear but has no bearing on whether the natural diets of mammals show phylogenetic signal and indicates nothing about how common PNC is among mammals.

Finally, DeSantis et al. (2018) rightly indicate that most PCMs assume that the residuals of y ~ x from an ordinary least squares regression are statistically independent (i.e., not phylogenetically autocorrelated). In cases where the x and y variables show no autocorrelation but the residuals of y do, ordinary least squares regression performs poorly (Revell, 2010). Herein, we demonstrate that when both x and y show high phylogenetic signal, the λ estimates for the residuals of y consistently indicate high phylogenetic signal (Figure 2). Given the scenario where both diet and tooth wear show high phylogenetic signal, as we demonstrate in our recent study, we are therefore justified in recommending the use of PCMs. Furthermore, Revell (2010) recommends fitting a phylogenetic generalized least squares model using maximum likelihood to simultaneously estimate λ and the regression coefficients. If λ is zero, PGLS converges on the ordinary least squares solution. Therefore, the application of a PCM, specifically PGLS, from the outset should not mislead researchers.

**Figure 2:**
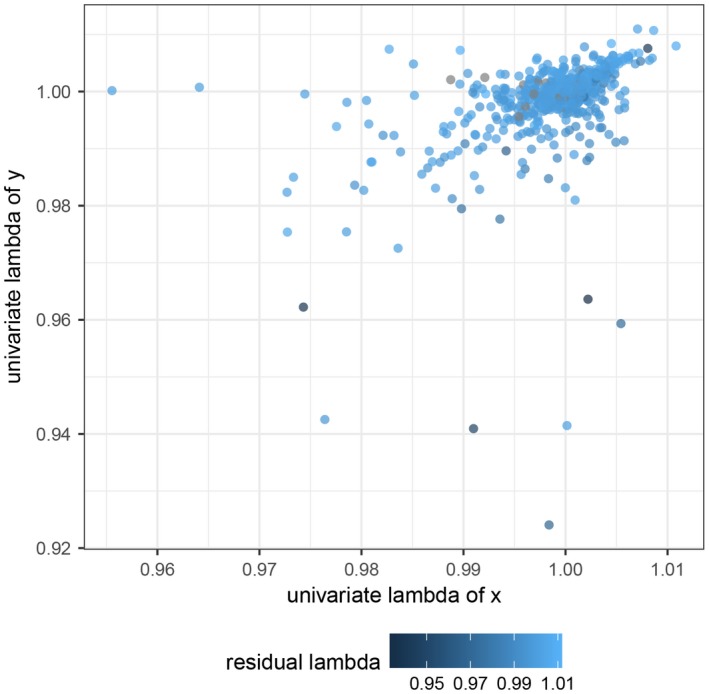
Phylogenetic signal in residuals from ordinary least squares regression is consistent high when phylogenetic signal in x and y is also high. Data were simulated using the fastBM function and tests of phylogenetic signal were performed using the phylosig function in the R package phytools (Revell, 2011)

In conclusion, we agree with DeSantis et al. (2018) that phylogenetic comparative methods need not be applied when their use is unnecessary or unjustified such as during a study of intraspecific dietary variation. However, we note that application of phylogenetic comparative methods (most notably phylogenetic generalized least squares) when phylogenetic signal is low will not lead researchers astray (Revell, 2010). Our recent study thoroughly describes the fact that phylogenetic signal can arise from descent with modification and PNC (see the Discussion section of our recent paper). We thus also agree with DeSantis et al. (2018) that phylogenetic signal in the context of tooth wear not be viewed as a mere inconvenient artifact. But we disagree with their implication that PCMs are therefore unnecessary. If authors are interested in interpreting p‐values from comparisons of species from disparate clades (e.g., camelids and bovids), they should have an understanding of the structure of their data as it pertains to phylogenetic signal.

## AUTHOR CONTRIBUTIONS

Danielle Fraser carried out the statistical analyses, interpreted the statistical analyses, and wrote the manuscript. Ryan J. Haupt wrote the manuscript. W. Andrew Barr helped design figures and wrote the manuscript.

## DATA ACCESSIBILITY

Data used in this reply are published in Mendoza, Janis, and Palmqvist (2002).
